# Pollen allergens do not come alone: pollen associated lipid mediators (PALMS) shift the human immue systems towards a T_H_2-dominated response

**DOI:** 10.1186/1710-1492-5-3

**Published:** 2009-10-22

**Authors:** Stefanie Gilles, Valentina Mariani, Martina Bryce, Martin J Mueller, Johannes Ring, Heidrun Behrendt, Thilo Jakob, Claudia Traidl-Hoffmann

**Affiliations:** 1 ZAUM - Center for Allergy and Environment, Division of Environmental Dermatology and Allergy Helmholz Center/TUM, Biedersteiner Str. 29, 80802 Munich, Germany; 2 Istituto dermopatico dell immacolata, Rome, Italy; 3 Julius-von-Sachs-Institute of Biosciences, Division of Pharmaceutical Biology, University of Würzburg, Würzburg, Germany; 4 Department of Dermatology and Allergy Biederstein, Technische Universität München, Munich, Germany; 5 Allergy Research Group, University Medical Center Freiburg, Freiburg, Germany

## Abstract

Pollen allergy is characterized by a T_H_2-biased immune response to pollen-derived allergens. However, pollen-exposed epithelia do not encounter pure allergen but rather a plethora of protein and non-protein substances. We demonstrated that pollen liberate lipids with chemical and functional similarities to leukotriens and prostaglandins - the pollen associated lipid mediators (PALMs). To date, two main groups of PALMs have been characterized: The immunostimulatory PALMs activating innate immune cells such as neutrophils and eosinophils, and the immunomodulatory E_1_-phytoprostanes blocking IL-12 production of dendritic cells, resulting in the preferential induction of T_H_2 responses. This article reviews our work in the field of PALMs and their effects on cells of the innate and adoptive immune system. From recent results a general picture starts to emerge in which PALMs (and possibly other pollen-associated substances) may - independently from protein allergens - propagate an overall T_H_2 favoring micromilieu in pollen exposed tissue of predisposed individuals.

## Background

Atopic diseases are characterized by a predominance of T helper cell type 2 (T_H_2) biased immune responses to environmental allergens. It is well established that allergen specific T_H_2 cells are the key orchestrators of allergic reactions, initiating and propagating inflammation through the release of a number of T_H_2 cytokines. While the importance of T_H_2 cells in allergy is well accepted, little is known about the mechanisms that control the initial T_H_2 polarization in response to exogenous allergens. While for some aeroallergens, foremost house dust mite Der p 1, several intrinsic T_H_2 adjuvant effects have been reported [1-3], most major pollen allergens seem to lack such characteristics.

A hallmark in the elucidation of adjuvant factors from pollen was the discovery that pollen release NADPH oxidases which increase reactive oxygen species in lung epithelium thereby promoting neutrophil recruitment and boosting allergic airway inflammation. In contrast, challenge with Amb a 1, the major ragweed allergen alone, did not result in robust airway inflammation [4].

As link between innate and adaptive immune system, dendritic cells (DCs) play a pivotal role in sensing environmental danger signals such as bacterial or viral products, and in mounting a T cell-mediated immune response against those potentially harmful invaders [5]. As professional antigen-presenting cells DCs reside in the periphery in an immature state, where they take up pathogens or allergens. Upon maturation, the cells undergo a series of phenotypic changes: while their capability to phagocytose antigen decreases, intracellular protein processing and presentation, as well as the expression of co-stimulatory markers are enhanced. The DCs acquire a migratory phenotype, serving their mission to transport the sampled antigen to the secondary lymphoid tissues. The trafficking of immature DCs to sites of inflammation and of mature DCs to the T cell area of secondary lymphoid organs is regulated by the expression of different chemokines and chemokine receptors [6].

In the defense against intracellular microbes or tumors, the key cytokine secreted by DCs is IL-12 [7], which skews T cell responses in the direction of T_H_1 [8]. IL-12 is induced by pathogen associated molecular patterns such as LPS or by T-cell derived signals such as IL-4 or CD40 ligation [9]. However, simultaneous presence of endogenous signals such as IL-10, TGF-β, corticosteroids, vitamin D_3_, or PGE_2 _can convert DC from T_H_1- to T_H_2-skewing antigen presenting cells [10]. Recent studies demonstrate that also exogenous factors such as lipids produced by parasites can modulate DC function for the purposes of evading host immunity [11].

Besides their well established role in host defence, DCs are also involved in hypersensitivity reactions against harmless environmental antigens, the allergens [12]. Indeed, evidence emerges that DCs are not only key players in allergic sensitization [13,14] but possibly even contribute to maintaining and shaping the immune response to allergens in already sensitized individuals [15,16]. Understanding the role of DCs in allergic sensitization has been hampered, however, by the fact that to date only very few signals have been identified that actively lead to a T_H_2 promoting DC phenotype [17,18].

We recently demonstrated that pollen, under physiologial exposure conditions, release not only allergens but also bioactive lipids. Among these are monohydroxylated derivatives of linoleic and linolenic acid [19] that resemble human Leucotriens and activate human neutrophils and eosinophils *in vitro*. We then extended these data on the impact of pollen associated lipid mediators on dendritic cell function. In brief, dinor isoprostanes (phytoprostanes) released from pollen grains under physiological conditions are able to inhibit the DC's production of IL-12 p70, and DC stimulated with aqueous pollen extracts or E_1_-phytoprostanes become T_H_2 skewing in mixed lymphocyte reaction. Additionally, DCs matured in the presence of aqueous pollen extracts respond by releasing T_H_2 attracting chemokines and aquire a distinct migratory phenotype. Finally, we could show that in a murine sensitization model, nasal instillation of OVA together with aqueous pollen extracts lead to a T_H_2 shift in draining lymph node T cells. Taken together, multiple lines of evidence imply that by modulating functions of the innate and adaptive immune system, PALMs add to creating a T_H_2 favoring, pro-allergic micromilieu.

## Pollen release lipid mediators - the PALMs

It is commonly accepted that in susceptible individuals, allergic sensitization results after allergens have been taken up by antigen-presenting cells residing in the barrier-forming epithelia like skin or airway mucosa. When investigating this allergic sensitization phase, most studies use purified allergen or allergen-extracts. Under physiological exposure conditions, however, pollen-derived allergens are not released alone, but rather in conjunction with pollen granules, starch grains and other, non-protein substances. One major constituent of pollen excine and exsudate are lipids which are essential in the plant fertilization process as they help the pollen tube to penetrate the stigma [20]. This prompted us to investigate the impact of the whole pollen grain on the human immune system. We recently demonstrated that upon hydration, pollen grains very rapidly release significant amounts of lipids- the so-called pollen-associated lipid mediators (PALMs) - that show structural and functional homology to eicosanoids [21]. Since arachidonic acid metabolites are well known to affect human innate and adaptive immune responses we were prompted to further investigate the effects of aqueous pollen extracts and their constituents.

## PALMs potently attract and activate PMN and eosinophils

The finding that pollen grains interact with cells of the human immune system was made by Siegel and Sherman as early as the seventies [22]. We were able to extend these observations by investigating the outcome of granulocyte - pollen interactions. Our data show that pollen grains (birch and grass) attract and activate neutrophils [23] and eosinophils [24] leading to the release of myeloperoxidase and eosinophilic cationic protein, respectively. Chemotactic activity seemed to be independent of protein allergen and could be demonstrated in aqueous pollen extracts (APE) as well as in total lipid extracts (Hexane-isopropanol extracts, HIP) and reverse phase extracts of HIP, enriched for mono-hydroxylated products of linoleic acid. Chemotaxis of Eosinophils was blocked by the LTB_4 _receptor antagonist LY293111, whilst APE-induced calcium influx in PMN was inhibited by pre-treatment with LTB_4 _and vice versa in cross-sensitization experiments. Interestingly, these effects seemed to be independent of the sensitization status of the donor and thus might occur in allergic and non-allergic individuals, further arguing for allergen-independent effects. Taken together, these findings indicate that, alongsinde the adaptive immune system, innate mechanisms may also contribute to the recognition of allergens within the respiratory tract.

## PALMs confer a T_H_2 promoting phenotype on DCs

Apart from their effects on neutrophils and eosinophils we investigated the impact of PALMs on human dendritic cells - the initiators of T cell responses. As model, we focused on human monocyte-derived dendritic cells (moDCs). Interestingly, exposure of moDCs with LPS-depleted aqueous birch pollen extracts (*Bet*.-APE) resulted in a selective upregulation of HLA-DR surface expression, while other maturation markers such as CD80, CD86, CD40 and CD83 were not modulated. On LPS-matured moDCs, *Bet.-*APE synergized with LPS in the up-regulation of all maturation markers tested. At a functional level, *Bet.-*APE stimulation of moDC resulted in an enhanced allostimmulatory activity as demonstrated by enhanced proliferative responses of naive allogeneic CD4^+ ^T cells. Importantly, *Bet*.-APE treatment of moDCs induced a dose dependent inhibition of the LPS or CD40L induced IL-12 p70 production, while IL-6, IL-10 and TNF-α production were not impaired. Thus, water soluble factors released from pollen grains are capable to selectively modulate various DC functions, including the inhibition of the key T_H_1 cytokine IL-12 p70 [25].

By means of gas chromatography-mass spectometry analysis of *Bet*.-APE, we demonstrate the presence of E_1_-, F_1_-, A_1_/B_1_-phytoprostanes in aqueous pollen extracts (see table 1) and show that E_1_-phytoprostanes - similar to *Bet*.-APE - dose-dependently inhibit the IL-12 p70 production while not affecting IL-6 production. Like in the case of *Bet.-*APE, pre-treatment of moDC with E_1_-phytoprostanes results in an increased IL-4/IFN-γ ratio in CD4^+ ^T cells after allogenic mixed lymphocyte reaction. Thus, PPE_1 _could be identified as one of the substances contained in *Bet.-*APE which mediate the T_H_2 polarizing capacity of moDCs [25].

**Table 1:** Concentrations of phytoprostanes in aqueous birch pollen extracts (modified from [25]).

	**Concentration in Bet.-APE (10 mg/mL) (nM)**	**Concentration (μg/g pollen)**
PPE_1_	543.6 +/- 41.1	17.72 +/- 1.34
PPF_1_	68.6 +/- 1.5	2.25 +/- 0.05
PPA_1_/B_1_	23.8.6 +/- 3.5	0.74 +/- 0.11

## Aqueous pollen extracts modulate chemokine/chemokine receptor expression and migratory capacity of DCs

Maturation of DCs results in substantial changes in the surface expression of T cell costimulatory molecules like HLA-DR, CD40, CD86 and CD80. Concomitantly, maturing DCs undergo distinct changes in the expression of chemokine receptors, licensing them to migrate towards chemokine gradients [26]. In a more recent study we therefore examined the effects of aqueous birch pollen extracts (*Bet*.-APE) on chemokine production, chemokine receptor expression and migratory capacity of moDCs [27]. Here we found that on immature DCs, *Bet*.-APE induced expression and function of CXCR4, which might be critical for directing DCs to lymphoid organs during allergic inflammation. Concomitantly, *Bet.-*APE reduced surface expression of CCR1 and CCR5, reflecting DC maturation and acquisition of a "pro-inflammatory" phenotype [26]. In addition, maturation of DCs with LPS in the presence of *Bet*.-APE impaired the LPS-induced production of the T_H_1 attracting chemokines CXCL10 and CCL5. Instead, the cells show an enhanced release of the "T_H_2" chemokine CCL22. The release of CCL17, a chemokine enhanced in atopic ekzema, was not significantly changed as compared to LPS treatment alone. At a functional level, *Bet.-*APE increased the capacity of LPS-matured DCs to migrate towards CXCL12 - as reflected by the enhanced expression of CXCR4 - and towards the lymph node homing chemokines CCL19 and CCL21. These effects of *Bet.-*APE depended on adenylyl cyclase and cAMP induction and strongly mimicked some key characteristics of PGE_2 _[28,29]. Finally, culture supernatants of DCs matured in the presence of LPS and *Bet*.-APE attracted T_H_2 cells in transwell chamber migration assays, while the capacity to recruit T_H_1 cells was reduced. This might imply that pollen-exposed DCs favor the maintainance of already established T_H_2 immune responses. Importantly, all effects summarized above were observed in DCs derived from monocytes of non-atopic donors. In our view this supports the concept of allergen-independent adjuvant effects of pollen shifting the primary immune response towards T_H_2 in susceptible individuals (see table 2).

**Table 2:** Summary of effects of PALMs on cells of the innate and adoptive immune system

**PMN**	**Eosinophils**	**Dendritic cells**
Chemotaxis*	Chemotaxis^#^	IL-12^¶↓^
Calcium influx*		T_H_2 bias in MLR^¶^
CD11b*↑	CD11b^#↑^	cAMPi^§↑^
Release of MPO*^↑^	Release of ECP^↑#^	CXCR4^§↑^
		CCR5, CCR1^§↓^
		CCL22^§↑^
		CXCL10, CCL5^↓§^

Arrows indicate an increase (↑) or decrease (↓).

References: *: Traidl-Hoffmann C et al., J Allergy Clin Immunol (2002); ^#^: Plötz SG et al., J Allergy Clin Immunol (2004); ^¶^: Traidl-Hoffmann C et al., J Exp Med (2005); ^§^: Mariani V et al., J Immunol (2007)

cAMP_i _= intracellular cyclic 5'-adenosine monophosphate; ECP = eosinophil cationic protein; MLR = mixed lymphocyte reaction.

## Factors from pollen lead to a preferential induction of T_H_2 responses *in vivo*

Only recently we were able to undermine our *in vitro *data by studies in a murine sensitization model [30]. OVA-specific CD4^+ ^T cells were adoptively transferred into BALB/c mice. Twenty-four hours later, mice were challenged by means of intranasal application of OVA in the absence or presence of *Bet.-*APE or phytoprostanes -E_1 _or -F_1_. Polarization of T-cell responses *in vivo *was analyzed in draining lymph node T cells. While intranasal instillation of phytoprostanes down-regulated both T_H_1 and T_H_2 cytokines, inhalation of *Bet.-*APE lead to a selective down-regulation of IFN-γ and an up-regulation of the T_H_2 cytokines IL-4, IL-5 and IL-13. This implies that water-soluble factors released from pollen might confer a T_H_2 polarizing capacity independently from phytoprostanes. The identification of those water-soluble substance(s) and dissecting their respective contributions to allergic sensitization or exacerbation should add to our general understanding of the mechanisms of pollen-induced allergy and might ultimately lead to the development of new therapeutic strategies.

**Figure 1:**
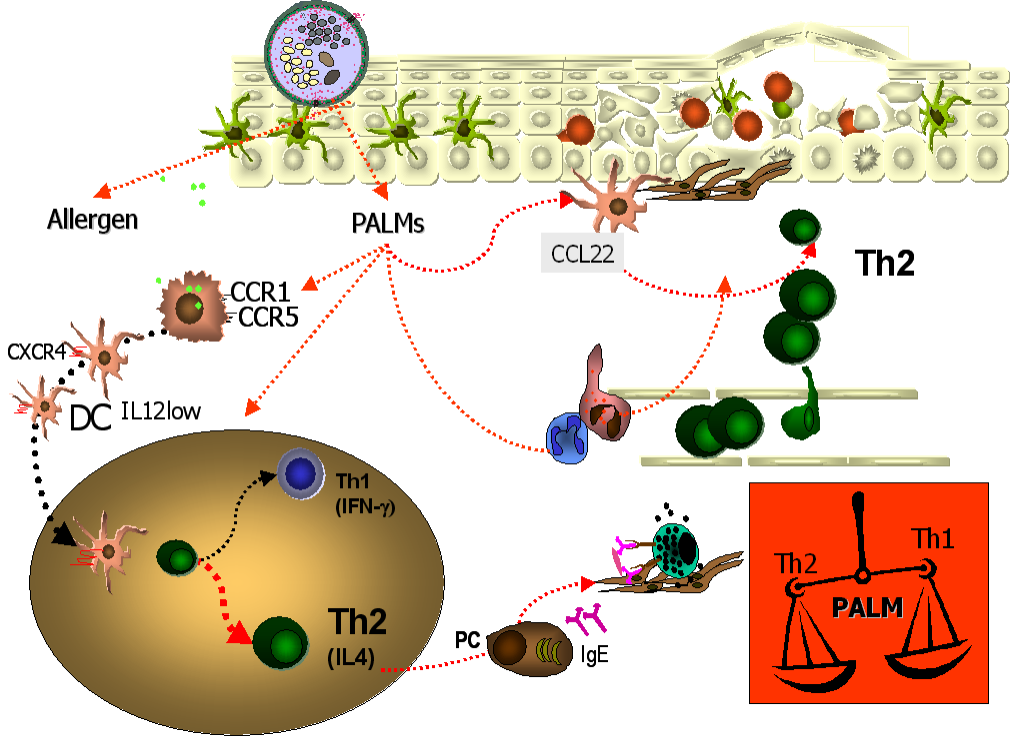
**Hypothetical model of a T_H_2 dominated adoptive immune response and local T_H_2 promoting micromilieu induced by pollen-associated lipid mediators**. When pollen grains are hydrated on the respiratory epithelia, they release allergens and eicosanoid lipids, the so-called pollen-associated lipid mediators (PALMs). Leucotrien-like PALMs have the potential to attract and activate innate cells like neutrophils and eosinophils, while prostaglandin-like PALMs, the phytoprostanes, and possibly other pollen-derived factors, can modulate the migratory and T helper cell polarizing capacities of resident dendritic cells. In addition, DCs exposed to PALMs might be induced to secrete chemokines which preferentially recruit further T_H_2 cells to the site of pollen exposure. Taken together, the possible effects of PALMs on both cells of the innate and the adoptive immune system might lead to a local microenvironment favoring T_H_2 responses. I FN-γ = interferon-γ; IL = interleukin; PC = plasma cell

In summary, pollen release regulatory mediators which might add to the generation of an overall T_H_2 promoting micro milieu. First, pollen provide signals for DCs to mature and acquire a migratory phenotype, preferentially priming type 2 T helper cell responses. The latter effect is partly mediated by E_1_-phytoprostanes, but other substances are likely to play a role. Secondly, PALMs might help to maintain an established T_H_2 response by preferential recruitment of T_H_2 cells and other inflammatory cells (neutrophils, eosinophils) to the site of pollen exposure (Figure 1) (see also table 2).

## Abbreviations

APE: aqueous pollen extract(s); Bet.-APE: aqueous birch pollen extracts; DC: dendritic cell; MoDC: monocyte-derived dendritic cell; OVA: ovalbumin; PALM: pollen-associated lipid mediator

## Competing interests

The authors declare that they have no competing interests.

## Authors' contributions

All authors contributed equally to the manuscript. All authors have read and approved the final manuscript.
